# Trajectories of Growth and Serum DHEAS and IGF-1 Concentrations in Girls With a History of Premature Adrenarche: Attenuation of the Phenotype by Adulthood

**DOI:** 10.3389/fendo.2018.00375

**Published:** 2018-07-10

**Authors:** Jani Liimatta, Pauliina Utriainen, Raimo Voutilainen, Jarmo Jääskeläinen

**Affiliations:** Department of Pediatrics, University of Eastern Finland and Kuopio University Hospital, Kuopio, Finland

**Keywords:** dehydroepiandrosterone sulfate, growth, insulin-like growth factor 1, menarche, pubarche

## Abstract

**Background:** It has been speculated that premature adrenarche (PA) could lead to unfavorable outcome, including shorter adult stature, but longitudinal follow-up data are insufficient.

**Methods:** This prospective case-control study included 30 PA and 42 control females who were born mostly full-term and appropriate for gestational age. They were examined first at the median age of 7.6 years and now at 18.1 years. Main outcome measures were height, body mass index (BMI), age at menarche, and serum dehydroepiandrosterone sulfate (DHEAS) and insulin-like growth factor 1 (IGF-1) concentrations.

**Results:** The PA and control females had comparable mean (standard deviation) adult height [167.2 (6.8) vs. 164.5 (5.1) cm, *P* = 0.059] and median (25th–75th percentiles) BMI [22.8 (21.1–28.9) vs. 21.6 (19.8–24.3) kg/m^2^, *P* = 0.068, respectively]. Adult heights were comparable with the mid-parental heights in both study groups. The PA females were taller than the controls until the age of 12 years and they lacked a distinct pubertal growth spurt. Serum DHEAS and IGF-1 concentrations did not differ between the PA and control groups at the age of 18 years. Median (range) age at menarche was significantly lower in the PA than control females [11.5 (9.5–15.0) vs. 13.0 (10.0–15.0), *P* = 0.001].

**Conclusions:** Although PA girls have advanced growth and earlier pubertal development together with a tendency to be more overweight, their height, BMI, and serum DHEAS and IGF-1 concentrations are comparable to those of their peers at the age of 18 years. Our findings indicate a benign outcome of PA in appropriate for gestational age -born females concerning adult height and adrenal androgen secretion.

## Introduction

Premature adrenarche (PA) is defined by increased levels of adrenal androgens before the age of 8 years in girls together with the presence of some hyperandrogenic clinical signs including adult-type body odor, comedones, acne, greasiness of hair and skin, and axillary or pubic hair (1, 2). The term premature pubarche (PP), by definition, refers to the appearance of pubic and/or axillary hair before the age of 8 years in girls. About half of the PA children have PP at the time of PA diagnosis in the Northern European Caucasian population (1, 3). The clinical presentation of PA is relatively well described. Compared with healthy peers, PA children have taller stature (3–5) and advanced bone age (BA) (3, 5–7) often associated with overweight (8, 9) and increased fat mass (10). They also tend to have decreased insulin sensitivity (9, 11) and higher serum insulin-like growth factor 1 (IGF-1) concentrations (5, 6, 12). These prepubertal findings have raised concerns that PA may lead to unfavorable outcome including shorter adult stature (7), but appropriate data on long-term outcome of PA are insufficient.

To date, studies investigating adolescent or adult PA subjects are limited (4, 13–19), especially those with a prospective setting (18, 19). Contrary to our cohort (5, 20), most of the previous studies have investigated only children with PP (13–19), some with high proportion of those being born small for gestational age (SGA) (15, 16, 18, 19). In three studies with mostly appropriate for gestational age (AGA) -born subjects, PA (4) or PP (14, 17) females have had earlier menarche and reduced pubertal growth (4, 14), but normal adult height, postpubertal body mass index (BMI), and late- or postpubertal dehydroepiandrosterone sulfate (DHEAS) concentrations (4, 14, 17). In our case-control cohort, mostly AGA-born PA females had higher pubertal height and BMI, and a higher percentage of them had reached menarche by the age of 12 years (20).

In this follow-up study, we evaluated females with a history of PA at the age of 18 years and aimed to investigate the following: (a) whether these currently young adult females have had earlier timing of menarche; (b) whether they have remained taller and more overweight than their peers or (c) if they have reduced adult height; and (d) whether they still manifest with exaggerated serum DHEAS and IGF-1 concentrations. Furthermore, we analyzed the trajectories in these anthropometric and biochemical parameters throughout the whole follow-up study.

## Subjects and methods

### Subjects and design

We have originally recruited 63 PA and 80 healthy age- and sex-matched control females to our follow-up cohort at the median age of 7.6 years between 2004 and 2006 (= baseline, prepuberty) (21). In the current study, we invited these females to attend a follow-up visit at the age of 18 years (= postpubertal examination), and altogether 23 PA and 42 control females participated. Additionally, we recruited all patients with a history of PA evaluated between 2000 and 2008 in our pediatric outpatient clinic at Kuopio University Hospital (*n* = 45) and, from this set, seven females were willing to participate. When compared to the PA females of our original cohort, additionally recruited females did not have any significant differences at baseline regarding to androgenic signs and anthropometric or biochemical variables. Thus, the total number of females in the current study was 72: 30 PA females and 42 controls, all Caucasian. Of these subjects, 17 PA and 30 control females had participated in a follow-up visit also at the age of 12 years (20).

PA definition and inclusion criteria for the PA group were at least one clinical sign of adrenal androgen action (adult-type body odor, comedones/acne, greasiness of hair/skin, axillary/pubic hair) before the age of 8 years together with an increased serum DHEAS concentration. As the PA girls were originally recruited on the clinical basis, some of them had their serum DHEAS concentration slightly below 40 μg/dL (1 μmol/L) at the baseline. However, all the PA females had distinct evidence of increased DHEAS secretion and in most of them (25/30, 83%), serum DHEAS concentration exceeded this “traditional” threshold level. Exclusion criteria for PA group were other causes of hyperandrogenism including central precocious puberty, congenital adrenal hyperplasia, androgen-producing tumors, and external exposure to androgens. None of the females reported any significant health issues or the use of long-term medication which would have affected their adrenal maturation, growth, or development.

Regarding to prepubertal and pubertal anthropometric or biochemical parameters, participating and non-participating females in each study group did not have any significant differences. Additionally, we inquired adult height and weight by posted questionnaires for those females who were unwilling to participate in the current follow-up visit. Altogether 29% of these non-attenders responded (12 PA and 11 control females) without any significant differences to the attending PA and control females.

This study was conducted in accordance with the Declaration of Helsinki. Research Ethics Committee of the Hospital District of Northern Savo approved the study protocol, and we obtained an informed consent from all participants and their parents.

### Clinical assessment

Birth data were collected from the medical records and were unavailable for 4 PA females. The parents were enquired upon their heights and timing of puberty (maternal age at menarche and paternal timing of the pubertal peak height velocity). These were subjective estimates given by the parents, but as both growth and puberty are systematically monitored in Finland until the end of puberty, individuals are well aware of their height and pubertal tempo. Paternal pubertal growth spurt was classified into three different categories as compared with that of their peers: early, on-time, and late. A difference more than one year from average indicated early or late timing of the paternal pubertal growth spurt. Paternal data were unavailable for 3 PA females. We used the Tanner method (22) to calculate corrected mid-parental height (cMPH): mid-parental height - 6.5 cm for females.

The methods for clinical assessment at prepubertal (5) and pubertal (20) age have been reported previously. At current evaluation at the age of 18 years, we performed anthropometric measurements between 8:00 and 9:00 a.m. after an overnight fast. Height was measured using a calibrated Harpenden stadiometer (Holtain Ltd., Crymych, UK) and recorded to the nearest 0.1 cm as the mean of three repeated measurements. Weight was measured with a calibrated electronic scale and recorded to the nearest 0.1 kg. We assessed waist circumference after expiration at mid-distance between the bottom of the rib cage and the top of the iliac crest, hip circumference horizontally to the floor at the maximum extension of the buttocks, and head circumference as the maximal fronto-occipital circumference. Waist, hip, and head circumferences were all recorded to the nearest 0.1 cm. BMI was calculated as the weight in kilograms divided by the square of height in meters. Following the WHO classification for adults (23), BMI cut-off points at the age of 18 years were 25 kg/m^2^ for being overweight and 30 kg/m^2^ for obesity. Corresponding to the adult thresholds, BMI standard deviation score (SDS) analogs at prepuberty and puberty were 1.16 for being overweight and 2.11 for obesity. To calculate all anthropometric SDSs, we used the current Finnish references (24, 25). Data on menarcheal age were obtained from medical records or by direct interview and recorded as precise as possible with the accuracy of 0.1 years. As pubertal development is systemically monitored in Finland and girls are well aware of their menarcheal timing, the data of age at menarche were considered reliable. All females were postpubertal according to the Tanner staging (26) evaluated by a trained physician (J.L.).

As PA has a high prevalence (27) and moderate BA advancement does not associate with predicted short adult height (28), current Finnish practice is to determine BA only for those PA children who present with PP or another extreme phenotype. Therefore, BA determination was not included in our study protocol to avoid unnecessary radiation exposure, especially for healthy controls and PA children without PP. However, BA determination was performed as part of a standard clinical evaluation in a subgroup of PP girls at prepubertal age (5).

### Longitudinal growth data

In Finland, height and weight are monitored systemically by trained primary care nurses, at least annually for every child until the end of secondary education. With the signed consents, we requested these measurements from public-school health care providers and proper data (data available and time interval between repeated measurements < 2 years) for further analyses (growth velocity-for-age) were achieved from 12 PA and 23 control females. Growth velocity (cm/year) was assessed by calculating the change of height derived from a combination of growth data measured by the investigator and school health care providers.

### Biochemical analyses

The analytical methods in prepuberty have been reported previously (5). At the age of 18 years, blood samples were drawn between 7:00 and 8:00 a.m. after an overnight fast and the serum samples were stored at −80°C until assayed. Serum DHEAS and IGF-1 concentrations were analyzed in duplicate using enzyme-linked immunosorbent assays with an ELx808 microplate reader (Biotek Instruments Inc., Winooski, VT) and specific kits (DHEAS, cat 1950, Alpha Diagnostic International, San Antonio, TX; IGF-1, cat E20, Mediagnost, Reutlingen, Germany). For DHEAS, the intra- and inter-assay coefficients of variation were 3.2 and 5.5%, respectively. For IGF-1, the corresponding values were 1.9 and 11.2%. DHEAS and IGF-1 SDSs in the PA females were calculated from the values of the control group and 0 SDS illustrates the mean of the control group.

### Statistical analyses

We performed all statistical analyses using the SPSS 24.0 software (IBM Corp., Armonk, NY, USA). The associations or statistical differences with *P* < 0.05 were considered significant. We analyzed the normality of the distributions for all continuous variables visually from the histograms and using the Shapiro-Wilk test. To compare the differences in continuous parameters between the study groups, we used the independent samples *t*-test for normally distributed variables and Mann-Whitney *U*-test for non-normally distributed variables. To analyze the differences between the study groups in categorical variables, we used the Fisher's exact test. To analyze age-dependent changes within each study group, we used either the paired samples *t*-test for anthropometric parameters or the Wilcoxon signed ranks test for biochemical variables.

## Results

Birth and parental data of the PA and control females are shown in Table 1, and characteristics of the study groups at baseline (prepuberty, median age 7.6 years), at puberty (median age 12.0 years), and at postpubertal examination (median age 18.1 years) are depicted in Table 2.

**Table 1:** Birth and parental data of the PA and control females.

	**PA, *n* = 30^a^**	**Control, *n* = 42**	***P***
**BIRTH DATA**			
Gestational age, weeks	40.1 (38.6–41.2)	40.1 (39.3–41.4)	0.705^b^
Birth length, cm	49.0 (48.0–51.0)	50.5 (49.8–52.0)	**0.045**^b^
Birth length SDS	−0.17 (1.06)	0.28 (0.89)	0.064
Birth weight, kg	3.53 (2.98–3.71)	3.56 (3.30–3.92)	0.126^b^
Birth weight SDS	−0.31 (−0.87–0.31)	−0.01 (-0.35–0.69)	0.064^b^
Preterm, yes, n (%)	2 (7.7)	4 (9.5)	1
SGA, yes, n (%)	1 (3.8)	1 (2.4)	1
**PARENTAL DATA**			
cMPH, cm	166.5 (162.0–168.8)	166.5 (161.5–168.6)	0.815^b^
Maternal age at menarche, years	12.0 (11.0–14.0)	13.0 (12.0–14.0)	0.065^b^
Early paternal pubertal growth spurt, yes, n (%)	7 (25.9)	1 (2.4)	**0.005**

Continuous variables are expressed as mean (standard deviation) and analyzed using the independent samples t-test, unless noted otherwise. Categorical variables are analyzed using the Fisher's exact test. Significant P-values are highlighted with bold.

a Birth data were available for 26 and paternal pubertal growth data for 27 PA females;

b *Continuous non-normally distributed variables are expressed as median (25th−75th percentiles) and analyzed using the Mann-Whitney U-test. cMPH, corrected mid-parental height; SGA, small-for-gestational age; SDS, standard deviation score; PA, premature adrenarche*.

**Table 2:** Characteristics of the PA and control females throughout the follow-up study.

	**PA, *n* = 30**	**Controls, *n* = 42**	***P***
**AT PREPUBERTY*^b^***			
Age, year	7.6 (6.8–8.0)	7.4 (6.8–8.0)	0.671^a^
Height, cm	130.5 (8.7)	124.7 (6.8)	**0.002**
Height SDS	0.92 (0.99)	−0.23 (0.84)	<**0.001**
Weight, kg	31.5 (26.5–35.8)	24.8 (22.1–29.3)	**0.001**^a^
BMI, kg/m^2^	17.1 (15.6–22.2)	16.0 (14.8–17.4)	**0.023**^a^
BMI SDS	0.79 (1.30)	0.10 (1.10)	**0.020**
DHEAS, μg/dL	75 (43–103)	33 (18–48)	<**0.001**^a^
IGF-1, ng/mL	198 (153–237)	137 (110–176)	<**0.001**^a^
**AT PUBERTY***^b^*			
Age, year	12.0 (12.0–12.2)	12.0 (12.0–12.1)	0.315^a^
Height, cm	161.4 (7.2)	152.9 (5.7)	<**0.001**
Height SDS	1.03 (1.05)	−0.13 (0.83)	<**0.001**
Weight, kg	55.3 (46.7–69.1)	43.6 (37.2–50.8)	<**0.001**^a^
BMI, kg/m^2^	20.9 (18.6–26.1)	19.2 (16.3–21.4)	**0.007**^a^
BMI SDS	0.95 (0.91)	0.11 (0.98)	**0.007**
Waist circumference, cm	67.5 (63.2–81.2)	62.7 (59.0–67.8)	**0.019**^a^
Waist-to-height, %	42.0 (39.5–49.0)	41.0 (38.0–45.0)	0.281^a^
Waist-to-hip, %	76.5 (71.6–81.1)	77.7 (75.1–79.9)	0.180^a^
**AT POSTPUBERTAL EXAMINATION**			
Age, year	18.1 (17.8–19.6)	18.1 (17.9–18.3)	0.950^a^
Height, cm	167.2 (6.8)	164.5 (5.1)	0.059
Height SDS	0.06 (1.20)	−0.42 (0.91)	0.059
Height-to-cMPH, %	100.6 (2.9)	99.4 (2.7)	0.071
Sitting height-to-height, %	53.9 (1.1)	53.6 (1.4)	0.300
Weight, kg	63.3 (59.2–82.5)	59.8 (54.7–65.7)	**0.014**^a^
BMI, kg/m^2^	22.8 (21.1–28.9)	21.6 (19.8–24.3)	0.068^a^
BMI SDS	0.94 (1.16)	0.38 (0.92)	0.068
Hip circumference, cm	94.1 (89.5–105.6)	92.3 (85.9–95.9)	0.094^a^
Hip-to-height, %	56.2 (52.6–62.2)	54.8 (53.9–58.2)	0.299^a^
Waist circumference, cm	75.5 (69.8–89.3)	72.1 (68.0–81.2)	0.059^a^
Waist-to-height, %	46.3 (41.5–51.8)	44.0 (41.5–48.8)	0.268^a^
Waist-to-hip, %	81.5 (77.4–84.7)	79.1 (76.3–84.6)	0.337^a^
Head circumference, cm	55.6 (1.7)	54.7 (1.4)	**0.016**
Head circumference-to-height, %	33.3 (32.5–34.2)	33.0 (32.3–34.0)	0.706^a^
DHEAS, μg/dL	262 (158–351)	214 (135–297)	0.067^a^
IGF-1, ng/mL	382 (353–507)	394 (323–468)	0.518^a^
Age at menarche, year	11.5 (11.0–12.3)	13.0 (12.0–14.0)	**0.001**^a^

Variables are continuous with normal distribution, expressed as mean (standard deviation), and analyzed using the independent samples t-test, unless noted otherwise. Significant p-values are higlighted with bold.

a Non-normally distributed continuous variables are expressed as median (25th−75th percentiles) and analyzed using the Mann-Whitney U-test;

b *Data were available for 17 PA and 30 control females. BMI, body mass index; cMPH, corrected mid-parental height; DHEAS, dehydroepiandrosterone sulfate; IGF-1, insulin-like growth factor 1; PA, premature adrenarche; SDS, standard deviation score*.

### Anthropometry and menarcheal timing

At the age of 18 years, the PA females had reached normal mean adult height which was slightly higher but statistically comparable with that of the control females (167.2 vs. 164.5 cm, *p* = 0.059; Table 2). The mean adult heights were close to the cMPHs in both study groups (no significant difference in either group). The median weight was higher in the PA than control females (63.3 vs. 59.8 kg, *p* = 0.014; Table 2). Although without a statistical significance, the median hip (94.1 vs. 92.3 cm, *p* = 0.094) and waist circumference (75.5 vs. 72.1 cm, *p* = 0.059), and BMI (22.8 vs. 21.6 kg/m2, *p* = 0.068) had a trend to be higher in the PA females (Table 2). The median hip-to-height, waist-to-height, and waist-to-hip ratios were comparable in the study groups. The distributions of normal weight, overweight, or obese subjects at the age of 18 years did not differ between the study groups (60/20/20% in the PA and 76/19/5% in the control females, *p* = 0.115). Although the prevalence of obesity was 4-fold in the PA females compared to the controls, the difference was not statistically significant in the present sample (*p* = 0.060). The mean head circumference was higher in the PA than control females, but the median height-corrected head circumferences were similar, and the sitting height-to-height –ratio did not differ between the study groups (Table 2). The median age at menarche was 1.5 years lower in the PA than control females (Table 2), ranging from 9.5 to 15.0 years in the PA, and from 10.0 to 15.0 years in the control group.

### Anthropometric trajectories

**Figure 1:**
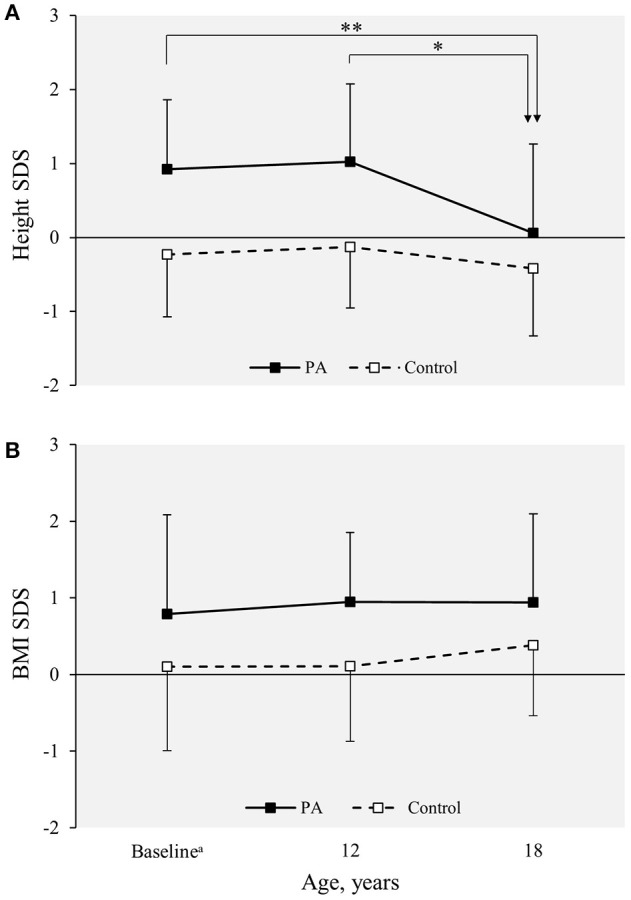
**(A)** Height and **(B)** BMI SDS in the PA (*n* = 30 at baseline and adulthood; *n* = 17 at the age of 12 years) and control females (*n* = 42 at baseline and adulthood; *n* = 30 at the age of 12 years) from prepuberty to adulthood. Values are expressed as mean (standard deviation). Statistical significance of the age-dependent changes within each group were analyzed using the paired samples *t*-test. ^**^p < 0.001; ^*^p < 0.01. BMI, body mass index; PA, premature adrenarche; SDS, standard deviation score.

A significant decrease in the mean height SDS of the PA females to the level corresponding with that of the control females occurred between the ages of 12 and 18 years (from 1.03 to 0.06, *P* = <0.001, Figure 1A). Mean BMI SDS remained constant during the whole follow-up study from prepuberty to adulthood without significant age-dependent changes (Figure 1B). At the age of 12 years, the height-to-adult height percentage was higher in the PA than control females (96.3 vs. 93.0%, *p* = 0.001).

**Figure 2:**
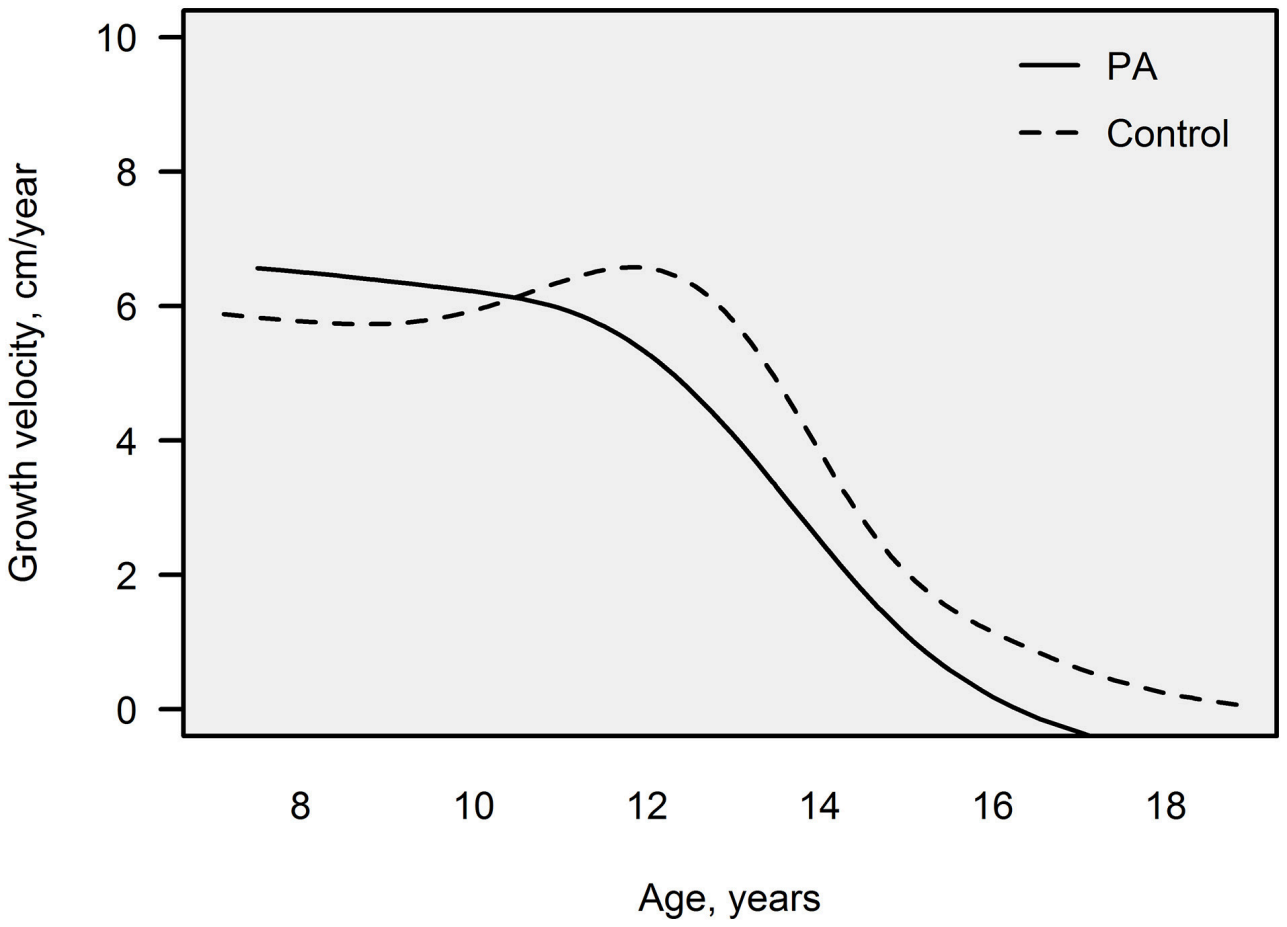
Growth velocity-for-age, illustrated as trend lines for the PA (*n* = 12) and control females (*n* = 23) whose detailed growth data were available. Growth velocity (cm/year) was assessed by calculating the change of height derived from a combination of growth data by the investigator and school health care providers. PA, premature adrenarche.

Growth velocity trend curves were drawn for the PA and control females whose detailed growth data were available (*n* = 12 for PA and 23 for control females). The shapes of these curves were different between the study groups: while the control females had a distinct pubertal increase in their height velocity, the PA females had a steadily declining growth velocity after 10 years of age (Figure 2).

### Biochemical findings

**Figure 3:**
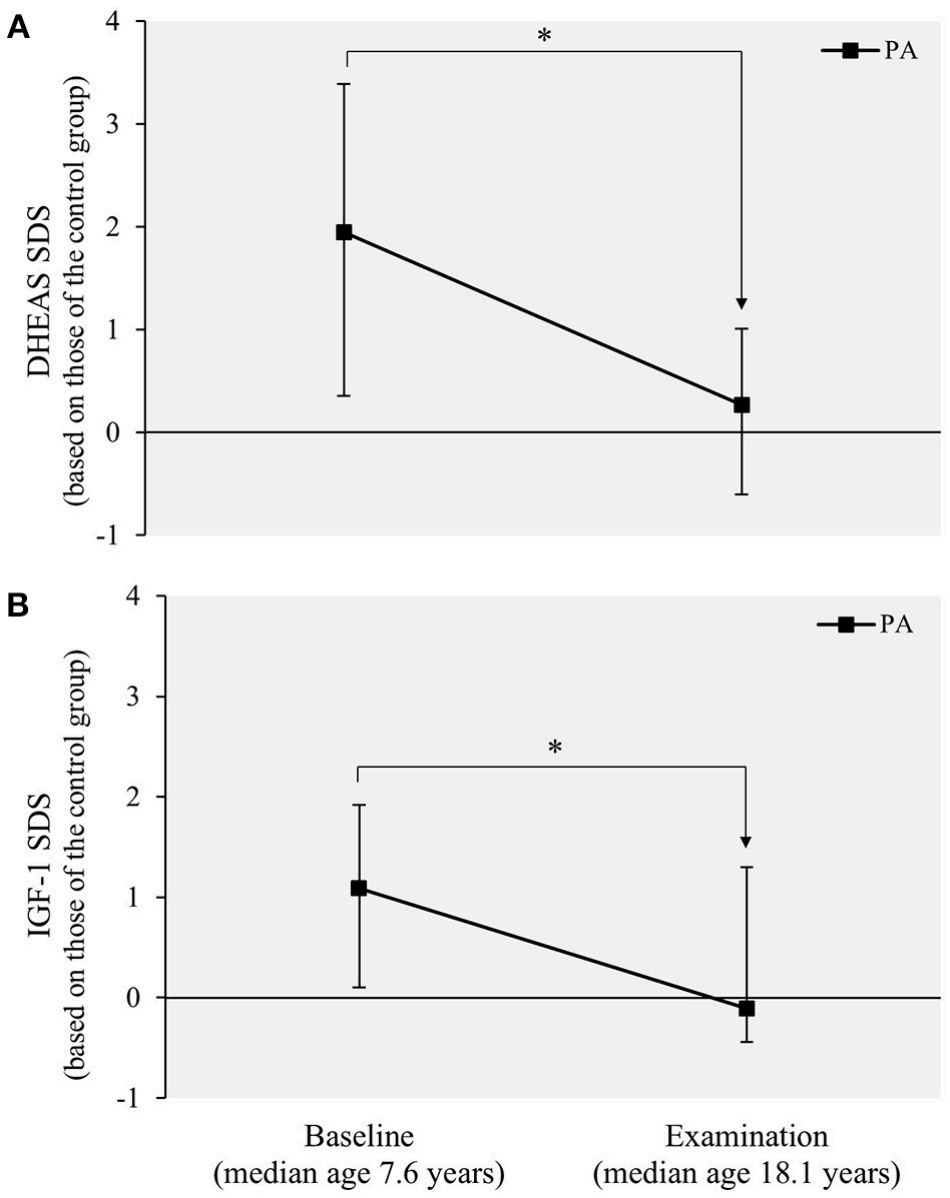
Serum **(A)** DHEAS and **(B)** IGF-1 concentrations in the PA females (*n* = 30) from prepuberty to adulthood. Values are expressed as median SDSs (25th−75th percentiles) based on those of the control females (*n* = 42). Statistical significance of the age-dependent change within each variable were analyzed using the Wilcoxon signed ranks test. ^*^P = < 0.001. DHEAS, dehydroepiandrosterone sulfate; IGF-1, insulin-like growth factor 1; PA, premature adrenarche; SDS, standard deviation score.

At the age of 18 years, DHEAS and IGF-1 concentrations did not differ significantly between the study groups (Table 2). When expressed as SDS values based on those of the control females, both median DHEAS and IGF-1 had significantly decreased in the PA females during the follow-up from 7.6 to 18.1 years (both *p* < 0.001; Figure 3).

### Birth and parental data

Although the median birth length (in cm) was slightly lower in the PA than control females, the mean birth length SDS did not differ significantly between the study groups (Table 1). Neither gestational age, birth weight, nor the frequencies of prematurity (gestational age <37 weeks) and SGA (birth weight or length < −2.0 SDS) differed between the study groups. While cMPH and maternal age at menarche did not differ between the PA and control females, the percentage of the fathers with early pubertal growth spurt was higher in the PA than control females (Table 1).

### Subgroups analyses between the PA females with and without PP

Altogether 16 of the participating PA females (53%) had presented with PP at the baseline. Birth data, parental parameters, or anthropometric and biochemical parameters at the age of 18 years did not differ between the PA subgroups with or without PP (data not shown). The median menarcheal age was 11.5 years in the PA females with PP and 12.0 in those without PP (*P* = 0.243).

## Discussion

In this case-control study, we investigated growth, timing of menarche, and serum DHEAS and IGF-1 concentrations prospectively in AGA-born PA and control females from prepuberty to adulthood. Our findings show that PA females have distinctive characteristics concerning growth, pubertal development, and serum DHEAS and IGF-1 concentrations. PA females have accelerated early prepubertal growth, they remain taller than the control females until late puberty, their pubertal growth spurt is attenuated, and they finally reach normal expected adult height. Similarly, prepubertally higher BMI persists in PA females until puberty, but their adult BMI is not necessarily different from that of the controls. Both serum DHEAS and IGF-1 concentrations are higher in PA than control females at prepuberty, but also these differences disappear during puberty leading to similar values by adulthood. In our population, PA females reach menarche at the median age of 11.5 years, which is approximately 1.5 years earlier than in control females.

Two previous studies have investigated PA children in a prospective setting (18, 19). The main finding in both studies was that the females with PP due to PA have slightly earlier pubertal development as their thelarche (18) and menarche (18, 19) occurred earlier than in the reference population. Although all PA females in these studies had PP and many of them were born SGA, the findings in our case-control cohort with mostly AGA-born PA females are similar with those found in the previous reports: the PA females had advanced pubertal development at the age of 12 years (20) and as our current study indicates, earlier menarcheal timing as well.

Regarding the growth pattern and final height in PA/PP children, our findings are well in line with previous studies. Taller stature (3, 4) and advanced BA (3, 6, 7) in prepubertal PA females has been reported in many cohorts, including ours (5). In one cross-sectional study, pre- and early pubertal PP females were taller than age- and pubertal stage -matched controls, but late and postpubertal PP and control females had similar height SDS (16). Comparable to our study, half of the PA subjects in a previous Finnish study had missing or reduced pubertal growth spurt (4). Normal adult height is a consistent finding in previous prospective (18, 19) and retrospective (4, 14) studies. Our study confirms in a prospective case-control setting that PA females use their growth potential earlier than their peers and reach normal expected adult height.

BMI and body composition in pubertal and postpubertal PP females have been studied in some previous cross-sectional studies (15–17). In one of these studies, BMI was higher in PP females than in controls at Tanner stages 2 and 3, but similar at Tanner stages 4 and 5 (15). In two other studies, PP females had similar BMI at pubertal (16) and postpubertal age (17), but higher fat mass and fat percentage at all pubertal stages (16) when compared to age-matched controls. In our cohort, the PA females were more overweight than the controls until mid-puberty, but their BMI at the age of 18 years did not differ significantly from that of the controls.

Previous studies have suggested that late or postpubertal DHEAS concentrations in PP females are normal as these values were comparable in 18 PP and 20 control females at late pubertal age (16), and between 27 PP and 25 control females at postpubertal age (17). In a Finnish study with AGA-born PA subjects, serum DHEAS concentrations were normal in PA adolescents when compared to reference values (4). Our findings are well in line with these previous studies showing that prepubertally higher DHEAS and IGF-1 concentrations in PA females moderate during puberty and are comparable to those of the controls at the age of 18 years.

In our previous report, we speculated that early factors in the life of PA females may contribute to the acceleration of growth and maturation (20). We believe that PA may rather be a by-product and an amplifying factor than the determinant in the process in which early accelerated growth and advanced pubertal development are adaptive mechanisms to the energy-rich environment and increased early weight gain, potentially enhanced by prenatal stress and smaller birth size. To support this hypothesis, increased weight gain in early childhood is associated with later childhood obesity, accelerated growth, and earlier pubertal development in healthy children (29). Second, rapid early weight gain (30–32), childhood obesity (33, 34), and several nutritional factors such as higher intake of animal (33) and vegetable protein (35), and low-fiber grain products (35) are independently associated with higher adrenal androgen secretion in healthy prepubertal children. In one of these studies, healthy children with highest DHEAS concentrations at the age of 7 years had gained most weight by the age of 4 and most height by the age of 7 years (32). Third, increased early weight gain is associated with PA in several cohorts (8, 36), including ours (5). In one of these studies, PA girls had adiposity rebound at the mean age of 3.7 years, which was significantly earlier than that of the controls (36). In another retrospective chart study with 89 PP children, weight SDS increased from birth to diagnosis in over 90% of the PP children, including those who had normal weight at PP diagnosis and those born AGA (8). In our cohort with mostly AGA-born PA females, weight-for-height increased from the age of one year (5). Finally, also lean PA females have increased fat mass (10, 16) and body adiposity modifies the clinical presentation of adrenarche (27). The findings in our cohort support this view as our AGA-born PA females had increased early weight gain together with advanced early growth, tall prepubertal and pubertal stature, and accelerated pubertal maturation but normal adult height and comparable BMI with their peers.

Our present study is the first longitudinal study on PA females which extends from prepuberty to adulthood and includes a healthy control group matched for age and gender. However, our study has some limitations. Participation percentage in the current postpubertal follow-up examination was relatively low, and we included seven additional PA females from our outpatient clinic to the original cohort to increase the number of PA subjects. Noteworthily, we did not detect any significant differences between the participating and non-participating females or between the PA females in our original cohort and the additionally recruited PA females. Thus, we believe that the participating subjects were representative samples of our original cohorts. We do not precisely know, by which instrument the heights were measured in schools. Although they are measured using standardized techniques with calibrated equipment, this inconsistency may yield a bias in the present study. Despite that we did not detect many statistically significant differences between the study groups, some variables in the PA females showed a trend toward higher values, especially BMI, waist circumference, and the prevalence of obesity. These differences might become statistically significant in larger cohorts. It should be noted that we did not assess metabolic profile or the features related to polycystic ovarian syndrome in this study; future evaluation will elucidate if our PA females have abnormalities relating to these aspects. Additionally, larger cohorts would allow one to properly analyze the differences between the PA phenotypes with and without PP.

In conclusion, growth advancement, more overweight body composition, and higher DHEAS and IGF-1 concentrations seems to be temporary features in prepubertal and adolescent AGA-born PA females. These clinical features attenuate during puberty leading to adult height, BMI, serum DHEAS, and IGF-1 concentrations that are comparable to peers. Timing of menarche in AGA-born PA females is earlier than in their peers. Our study suggests the benign nature of PA in AGA-born females. However, the limitations in the present study in relation to statistical power due to small sample size may hide some differences between the study groups. Further studies are needed to confirm these findings and to clarify body composition, metabolic profile, and the features of polycystic ovarian syndrome in adult females with a history of PA.

## Author contributions

PU, RV, and JJ conceived the project and designed the study. JL and PU performed collection and handling of the data. JL and JJ analyzed the data. All the authors discussed the data and accepted the final draft; JL wrote the manuscript with contributions from all the authors.

### Conflict of interest statement

The authors declare that the research was conducted in the absence of any commercial or financial relationships that could be construed as a potential conflict of interest.

## Abbreviations

AGA: appropriate for gestational age
BA: bone age
BMI: body mass index
cMPH: corrected mid-parental height
DHEAS: dehydroepiandrosterone sulfate
IGF-1: insulin-like growth factor 1
PA: premature adrenarche
PP: premature pubarche
SGA: small for gestational age
SDS: standard deviation score.

## References

[1] Utriainen P Laakso S Liimatta J Jääskeläinen J Voutilainen R. Premature adrenarche–a common condition with variable presentation. Horm Res Paediatr. (2015) 83:221–31. 10.1159/000369458 25676474

[2] Voutilainen R Jääskeläinen J. Premature adrenarche: etiology, clinical findings, and consequences. J Steroid Biochem Mol Biol. (2015) 145:226–36. 10.1016/j.jsbmb.2014.06.004 24923732

[3] Voutilainen R Perheentupa J Apter D. Benign premature adrenarche: clinical features and serum steroid levels. Acta Paediatr Scand. (1983) 72:707–11. 6227200 10.1111/j.1651-2227.1983.tb09798.x

[4] Pere A Perheentupa J Peter M Voutilainen R. Follow up of growth and steroids in premature adrenarche. Eur J Pediatr. (1995) 154:346–52. 7641763

[5] Utriainen P Voutilainen R Jääskeläinen J. Girls with premature adrenarche have accelerated early childhood growth. J Pediatr. (2009) 154:882–7. 10.1016/j.jpeds.2008.12.038 19230905

[6] Sopher AB Jean AM Zwany SK Winston DM Pomeranz CB Bell JJ. Bone age advancement in prepubertal children with obesity and premature adrenarche: possible potentiating factors. Obesity (2011) 19:1259–64. 10.1038/oby.2010.305 21311512 PMC3637026

[7] Gurnurkar S Arheart KL Messiah SE Mankodi A Carrillo A. Skeletal maturation and predicted adult height in children with premature adrenarche. J Pediatr Endocrinol Metab. (2014) 27:69–74. 10.1515/jpem-2013-0199 23959660

[8] Neville KA Walker JL. Precocious pubarche is associated with SGA, prematurity, weight gain, and obesity. Arch Dis Child. (2005) 90:258–61. 10.1136/adc.2004.053959 15723910 PMC1720316

[9] Utriainen P Jääskeläinen J Romppanen J Voutilainen R. Childhood metabolic syndrome and its components in premature adrenarche. J Clin Endocrinol Metab. (2007) 92:4282–5. 10.1210/jc.2006-2412 17698912

[10] Cebeci AN Tas A. Higher body fat and lower fat-free mass in girls with premature adrenarche. J Clin Res Pediatr Endocrinol. (2015) 7:45–8. 10.4274/jcrpe.1525 25800475 PMC4439891

[11] Vuguin P Linder B Rosenfeld RG Saenger P DiMartino-Nardi J. The roles of insulin sensitivity, insulin-like growth factor I (IGF-I), and IGF-binding protein-1 and−3 in the hyperandrogenism of African-American and Caribbean Hispanic girls with premature adrenarche. J Clin Endocrinol Metab. (1999) 84:2037–42. 10372707 10.1210/jcem.84.6.5722

[12] Silfen ME Manibo AM Ferin M McMahon DJ Levine LS Oberfield SE. Elevated free IGF-I levels in prepubertal Hispanic girls with premature adrenarche: relationship with hyperandrogenism and insulin sensitivity. J Clin Endocrinol Metab. (2002) 87:398–403. 10.1210/jcem.87.1.8143 11788683

[13] Miller D Emans SJ Kohane I. Follow-up study of adolescent girls with a history of premature pubarche. J Adolesc Health. (1996) 18:301–5. 8860795 10.1016/1054-139X(95)00234-J

[14] Oron T Lebenthal Y de Vries L Yackobovitch-Gavan M Phillip M Lazar L. Interrelationship of extent of precocious adrenarche in appropriate for gestational age girls with clinical outcome. J Pediatr. (2012) 160:308–13. 10.1016/j.jpeds.2011.08.009 21907353

[15] Ibáñez L Potau N Zampolli M Street ME Carrascosa A. Girls diagnosed with premature pubarche show an exaggerated ovarian androgen synthesis from the early stages of puberty: evidence from gonadotropin-releasing hormone agonist testing. Fertil Steril. (1997) 67:849–55. 9130889 10.1016/s0015-0282(97)81396-9

[16] Ibáñez L Ong K de Zegher F Marcos MV del Rio L Dunger DB. Fat distribution in non-obese girls with and without precocious pubarche: central adiposity related to insulinaemia and androgenaemia from prepuberty to postmenarche. Clin Endocrinol. (2003) 58:372–9. 10.1046/j.1365-2265.2003.01728.x 12608944

[17] Meas T Chevenne D Thibaud E Léger J Cabrol C Czernichow P. Endocrine consequences of premature pubarche in post-pubertal Caucasian girls. Clin Endocrinol. (2002) 57:101–6. 10.1046/j.1365-2265.2002.01579.x 12100077

[18] de Ferran K Paiva IA dos Santos Garcia L de Pinho Gama M Guimarães MM. Isolated premature pubarche: report of anthropometric and metabolic profile of a Brazilian cohort of girls. Horm Res Paediatr. (2011) 75:367–73. 10.1159/000324107 21464553

[19] Ibáñez L Jimenez R de Zegher F. Early puberty-menarche after precocious pubarche: relation to prenatal growth. Pediatrics (2006) 117:117–21. 10.1542/peds.2005-0664 16396868

[20] Liimatta J Utriainen P Voutilainen R Jääskeläinen J. Girls with a history of premature adrenarche have advanced growth and pubertal development at the age of 12 years. Front Endocrinol. (2017) 8:291. 10.3389/fendo.2017.00291 29163361 PMC5671637

[21] Utriainen P Voutilainen R Jääskeläinen J. Continuum of phenotypes and sympathoadrenal function in premature adrenarche. Eur J Endocrinol. (2009) 160:657–65. 10.1530/EJE-08-0367 19151133

[22] Tanner JM Goldstein H Whitehouse RH. Standards for children's height at age 2 to 9 years allowing for height of parents. Arch Dis Child. (1970) 45:755–62. 5491878 10.1136/adc.45.244.755 PMC1647404

[23] World Health Organization (WHO) Obesity: preventing and managing the global epidemic. Report of a WHO consultation. World Health Organ Tech Rep Ser. (2000) 894:1–253. Available online at: http://www.who.int/nutrition/publications/obesity/WHO_TRS_894/en/11234459

[24] Saari A Sankilampi U Hannila ML Kiviniemi V Kesseli K Dunkel L. New Finnish growth references for children and adolescents aged 0 to 20 years: length/height-for-age, weight-for-length/height, and body mass index-for-age. Ann Med. (2011) 43:235–48. 10.3109/07853890.2010.515603 20854213

[25] Sankilampi U Hannila ML Saari A Gissler M Dunkel L. New population-based references for birth weight, length, and head circumference in singletons and twins from 23 to 43 gestation weeks. Ann Med. (2013) 45:446–54. 10.3109/07853890.2013.803739 23768051

[26] Marshall WA Tanner JM. Variations in pattern of pubertal changes in girls. Arch Dis Child. (1969) 44:291–303. 5785179 10.1136/adc.44.235.291 PMC2020314

[27] Mäntyselkä A Jääskeläinen J Lindi V Viitasalo A Tompuri T Voutilainen R. The presentation of adrenarche is sexually dimorphic and modified by body adiposity. J Clin Endocrinol Metab. (2014) 99:3889–94. 10.1210/jc.2014-2049 25029425

[28] DeSalvo DJ Mehra R Vaidyanathan P Kaplowitz PB. In children with premature adrenarche, bone age advancement by 2 or more years is common and generally benign. J Pediatr Endocrinol Metab. (2013) 26:215–21. 10.1515/jpem-2012-0283 23744298

[29] Dunger DB Ahmed ML Ong KK. Early and late weight gain and the timing of puberty. Mol Cell Endocrinol. (2006) 254–255:140–5. 10.1016/j.mce.2006.04.003 16824679

[30] Ong KK Potau N Petry CJ Jones R Ness AR Honour JW. Opposing influences of prenatal and postnatal weight gain on adrenarche in normal boys and girls. J Clin Endocrinol Metab. (2004) 89:2647–51. 10.1210/jc.2003-031848 15181036

[31] Remer T Manz F. Role of nutritional status in the regulation of adrenarche. J Clin Endocrinol Metab. (1999) 84:3936–44. 10566631 10.1210/jcem.84.11.6093

[32] Mericq V Pereira A Uauy R Corvalan C. Early BMI gain and later height growth predicts higher DHEAS concentrations in 7-year-old Chilean children. Horm Res Paediatr. (2017) 87:15–22. 10.1159/000452885 27974716

[33] Shi L Wudy SA Buyken AE Hartmann MF Remer T. Body fat and animal protein intakes are associated with adrenal androgen secretion in children. Am J Clin Nutr. (2009) 90:1321–8. 10.3945/ajcn.2009.27964 19793857

[34] Corvalan C Uauy R Mericq V. Obesity is positively associated with dehydroepiandrosterone sulfate concentrations at 7 y in Chilean children of normal birth weight. Am J Clin Nutr. (2013) 97:318–25. 10.3945/ajcn.112.037325 23283497 PMC3545681

[35] Mäntyselkä A Jääskeläinen J Eloranta AM Väistö J Voutilainen R Ong K Associations of lifestyle factors with serum dehydroepiandrosterone sulfate and insulin-like growth factor-1 concentration in prepubertal children. Clin Endocrinol. (2017) 88:234–42. 10.1111/cen.13511 PMC6195184 29112780

[36] Marakaki C Karapanou O Gryparis A Hochberg Z Chrousos G Papadimitriou A. Early adiposity rebound and premature adrenarche. J Pediatr. (2017) 186:72–77. 10.1016/j.jpeds.2017.03.058 28457524

